# Factor Analysis of the Prediction of the Postpartum Depression Screening Scale

**DOI:** 10.3390/ijerph16245025

**Published:** 2019-12-10

**Authors:** Mei Cai, Yiming Wang, Qian Luo, Guo Wei

**Affiliations:** 1 School of Management Science and Engineering, Nanjing University of Information Science & Technology, Nanjing 210044, China; 18645809620@163.com; 2 Business School, Nanjing University of Information Science & Technology, Nanjing 210044, China; luoqian0131@163.com; 3 Department of Mathematics and Computer Science, University of North Carolina at Pembroke, Pembroke, NC 28372, USA; guo.wei@uncp.edu

**Keywords:** postpartum depression (PPD), postpartum depression screening scale (PDSS), decision tree (DT), factor analysis, prediction

## Abstract

Postpartum depression (PPD), a severe form of clinical depression, is a serious social problem. Fortunately, most women with PPD are likely to recover if the symptoms are recognized and treated promptly. We designed two test data and six classifiers based on 586 questionnaires collected from a county in North Carolina from 2002 to 2005. We used the C4.5 decision tree (DT) algorithm to form decision trees to predict the degree of PPD. Our study established the roles of attributes of the Postpartum Depression Screening Scale (PDSS), and devised the rules for classifying PPD using factor analysis based on the participants’ scores on the PDSS questionnaires. The six classifiers discard the use of PDSS Total and Short Total and make extensive use of demographic attributes contained in the PDSS questionnaires. Our research provided some insightful results. When using the short form to detect PPD, demographic information can be instructive. An analysis of the decision trees established the preferred sequence of attributes of the short form of PDSS. The most important attribute set was determined, which should make PPD prediction more efficient. Our research hopes to improve early recognition of PPD, especially when information or time is limited, and help mothers obtain timely professional medical diagnosis and follow-up treatments to minimize the harm to families and societies.

## 1. Introduction

In modern society, new mothers face various challenges that accompany adjusting to a newborn, such as sleep problems, newly added duties, or physical discomfort. Postpartum depression (PPD), a severe form of mental illness caused by pregnancy and childbirth, has also become more prevalent. Research data show that one in nine new mothers suffer from PPD [1]. It has also been established that the proportion of new mothers who are affected by PPD will increase as long as this illness remains undetected [2]. PPD has furthermore been a social problem for some time and healthcare professionals have not paid enough attention to this. Because of these historical reasons, new mothers and their families are often neglected and do not receive necessary evaluation and treatment.

During pregnancy, women at risk of depression are more likely to suffer preeclampsia, preterm delivery, and low birth weight infants [3]. Following delivery, women at risk of depression show a tendency to be irresponsible and feel sad, hopeless, empty, or overwhelmed. In some extreme cases, a new mother might even harm herself or her baby.

PPD also has effects on the infants of the affected mothers in that their babies may not receive the necessary and effective care. This can even last into the child’s adulthood. O’Higgins [4] conducted longitudinal research on the first year of an infant’s life and found that when women at risk of depression failed to bond well with their infant in the first month, it could lead to separation from their infants. Field [5] compared two groups of infants, one comprising infants of depressed mothers and the other infants of ordinary mothers, and found that infants of depressed mothers were subdued in their social lives, even remaining inactive when they participated in a still-face activity. Moreover, babies of mothers suffering from PPD may have problems with sleeping and eating behaviors when they grow up.

Without treatment, the effects of PPD can last for months or even years. Fortunately, most women suffering from PPD can recover if they receive timely treatment [6]. Early detection of PPD is essential to allow for enough time for further evaluation, treatment and support [7]. Success with regard to the screening, diagnosis, and treatment of depression will benefit both women suffering from PPD and their families. Many countries have already implemented mandatory screening for PPD, such as the Australian Government which has been implementing PPD screening for more than a decade. Organizations such as the Association of Women’s Health, Obstetric and Neonatal Nurses (AWHONN) and the United States Preventive Services Task Force (USPSTF) recommend that pregnant women, new mothers, and newborns have routine screening for depression. The aim of our study is to use in-depth information to establish the role of attributes in the Postpartum Depression Screening Scale (PDSS), and to determine the rules for classifying PPD through factor analysis. We used 586 samples based on the PDSS collected from Robeson County in North Carolina during 2002–2005. An important contribution of this paper is that it provides a tangible pregnancy database that can help to verify the efficiency of the rules for classifying PPD. We hope our research will help to minimize the harm to families and societies.

The remainder of this paper is organized as follows. Section 2 presents a brief review of the related works. Section 3 describes the classifications based on the C4.5 decision tree (C4.5 DT) algorithm for PPD prediction. Section 4 performs the performance evaluation and the analysis of the results of the proposed classifiers. Section 5 discusses the results from Section 4 and provides explanations and comparisons. Section 6 concludes the study, highlights the limitations of the study, and points out a future research direction.

## 2. Related Works

The effectiveness of screening to identify PPD has substantial relevance for the selection of screening instruments [8]. The Beck Depression Inventory (BDI) [9], the Edinburgh Postnatal Depression Scale (EPDS) [10], the Postpartum Depression Screening Scale (PDSS) [11], and the Postpartum Depression Predictors Inventory-Revised (PDPI-R) [12] are common depression instruments. The BDI has been the most frequently and widely used instrument and has achieved great success. In 1996, Beck, Steer and Brown published the Beck Depression Inventory-II (BDI-II) [13] that designed the screening instruments whose criteria were selected from the American Psychological Association’s (APA) Diagnostic and Statistical Manual for Mental Disorders, Fourth Edition. The EPDS, which is a self-report instrument, was designed to specifically screen for postpartum depression. The EPDS used a Likert-type format for responses. PDPI-R has been increasingly studied in Italy, Korea, Japan, Mexico, Spain and Portugal [12]. Beck and Gable [2,14] compared the PDSS with the EPDS and the BDI-II. They employed psychometric testing and concluded that the PDSS is valuable for routine screening of new mothers.

In addition to the above screening methods, other methods have been proposed. Moreira and Rodrigues [15] proposed an improved algorithm using artificial intelligence (AI) to predict the risk of postpartum depression during pregnancy through biomedical and sociodemographic data analysis. Gaillard and Le Strat [16] studied PPD among mothers of middle-class communities and found that several key factors affected the degree of PPD, such as socio-demographic, psychosocial, and obstetrical risk.

**Figure 1:**
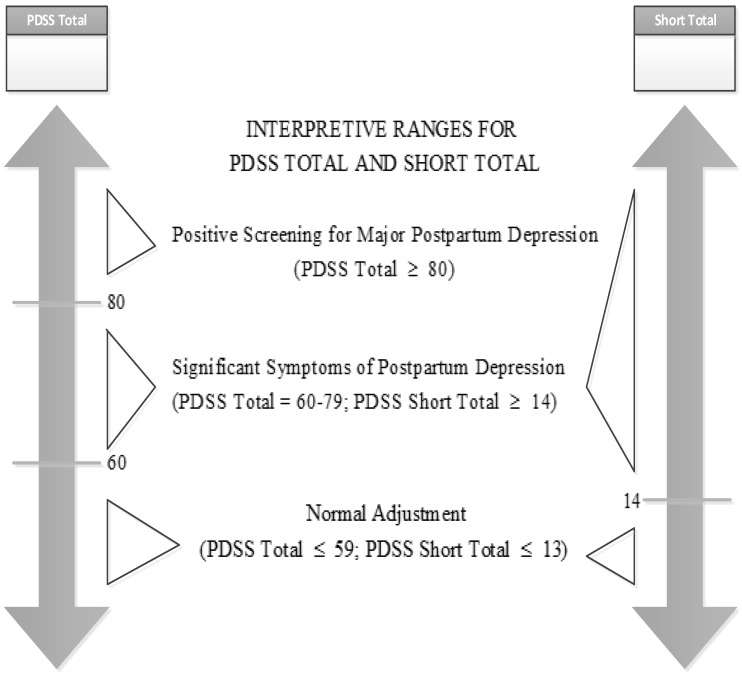
Interpretive ranges for the Postpartum Depression Screening Scale (PDSS) Total and Short Total.

The PDSS is a widely used screening instrument that is measured on a 35-item Likert response scale. The screening instrument is divided into seven dimensions with each dimension composed of five items. The seven dimensions are Sleeping/Eating Disturbances, Anxiety/Insecurity, Emotional Lability, Cognitive Impairment, Loss of Self, Guilt/Shame, and Contemplating Harming Oneself. On completing the scale, a mother is asked to select a label from (1) to (5) to reflect her degree of disagreement or agreement, where (1) means strongly disagree and (5) means strongly agree. The PDSS questionnaire consists of two parts: the first part focuses on the respondent’s demographics, gender, age, education level, etc. (named “individual demographics”); while the second part comprises the investigation content itself (named “symptom descriptions”). The second part of the PDSS is further divided into a short form and a full form. For the short form, items one through seven must be completed (each item is a question about the symptom description. In this paper, we view an item as an attribute of a multi-attributes decision problem). The sum of the scores in the short form delivers the score of the short total. For the full form of the PDSS, several boxes are arranged in columns labeled with the names of the seven PDSS symptom related scales. The respondent is asked to circle the answer which best describes how she has felt over the past two weeks. The circled response for each item is then transferred to the corresponding box printed in the same row. The scores are tallied to get each symptom scale total, and the sum of the symptom scales subsequently gives the full PDSS Total score. The degree of a mother’s PPD can be assessed according to the ranges for the PDSS Total and Short Total (see Figure 1).

Well known depression instruments vary in aspects of their item content [2], since each one reflects different instrument developers’ conceptual definitions of PPD. This difference in understanding of PPD naturally gives rise to different screening factors. Most research is based on statistical analyses and prefer to use statistical tools to obtain causal links between the attributes and the PPD degree. Among existing research, confirmatory factor analysis and standardized weight models are often used [17].

The selection of factors in a questionnaire is of crucial importance and varies across different depression instruments. Studies about the selection of factors, however, have seldom been conducted. Generally, questionnaires are vital to draw accurate information from respondents. From previous studies about PPD screening, it becomes clear that the first part of the questionnaire, which focuses on the respondent’s individual demographics, is underutilized. In studies of other diseases, much more attention is paid to the respondent’s information. For example, when diagnosing and managing adolescent depression, demographics is an important factor [18]. Duggan and Molina [19] studied the differences in socioeconomic variables and knowledge of screening practices and concluded that community-level factors in medically underserved areas may influence screening practices. Amuchastegui and Hur [20] included demographics as a major criterion of improvement to the screening process for infective endocarditis (IE). Chen and Cross [21] found women who lack social support, have an unstable economic status, and those experiencing acculturation were more likely to suffer depression. The first point of our research is that more attention should be paid to the information contained in the respondent’s demographics, i.e., gender, age, education level, etc. We attempt to reveal the role of such information when the PDSS is used as a screening instrument.

Additionally, once both parts (individual demographics and symptom description) are considered as criteria in the process of PPD prediction, a new prediction rule may be needed or the old one may need to be modified. We need to verify the efficiency of the combination of these two parts. More in-depth research that focuses on the deficiency of the short form and full form to predict the degree of PPD should be undertaken. Many studies only utilize the second part and treat it as the whole, neglecting the first part and disregarding the interactions among them. The score from the short form and the score from the full form are applied as the only criteria to predict PPD. PPD is subsequently simplified and vital information is neglected. The second aspect of our research is that more attention needs to be paid to the relationship and interactions of all factors contained in the questionnaire. We endeavor to find an effective way to predict PPD when the PDSS is used as a screening instrument.

## 3. Method

Our study is based on a collection of PPD questionnaires responded by 586. They were between 14–42 years old and came from Robeson County in North Carolina, covering all ethnic groups.

### Classification

In the following, the main concepts used in our model are described: 

Assume a set of case databases that contains n instances $Ins:=\left\{u_{1},u_{2},u_{3},\cdots ,u_{n}\right\}$, and attribute sets $At:=\left\{A_{1},\;A_{2},\;A_{3},\;\cdots A_{m}\right\}.$ The attribute $A_{p}$ is associated to a group of attribute values $A_{p}:=\{v_{1p},v_{2p},\cdots ,v_{np})$ of instance $u_{j}^{}$. These instances are classified into a certain category and the category set is represented as $Ca:=\left\{C_{1},C_{2},C_{3},\cdots ,C_{h}\right\}$.

In the training phase, we had to determine the rules for classifying data according to attributes. We use decision trees to represent the conceptual structure or relation of these attributes to corresponding categories. The decision tree can be used to visually and explicitly represent the data. A decision tree with a flowchart-like structure has leaves and branches. Each tree node is labeled with an attribute variable that produces branches for each value [22].

The decision tree is represented as follows:

- Each internal node tests a decision attribute;
- Each branch corresponds to a decision attribute value;
- Each leaf node assigns a category.

We use the C4.5 decision tree algorithm to form the decision tree. While there are several kinds of algorithms that can be utilized to form a decision tree, we adopted the C4.5 DT, which is a widely used classifier, based on the branch test. The main idea is that the root of the decision tree should be the most informative attribute. Information entropy is an index used to identify the most informative attribute. We expand the tree into branches associated with all possible focal elements of this attribute. For each branch, the free attribute with maximum information gain ratio will be the next node, from one level to the next, until the tree reaches the maximum specified depth or the maximum class probability reaches the given threshold probability [23].

First, we introduce five important concepts.

**Definition** **1.** *Entropy [24], E(S), is defined as:*

(1) $$E\left(S\right)=-\underset{x\in X}{\sum }p\left(x\right)log_{2}p\left(x\right)$$

*where*

S *—The current set for which entropy is being calculated;*
X *—Set of classes in* S *;*
p(x)*—The proportion of the number of elements in class X to the number of elements in set*S.

Entropy is a very common measure which is utilized to measure the amount of uncertainty in the set S.

**Definition** **2.** *For the ith attribute, the expected entropy [25], $EE\left(A_{i},S\right),$ is:*

(2) $$EE\left(A_{i},S\right)=\underset{A_{i}\in X}{\sum }p\left(A_{i}\right)E\left(S\right)$$

**Definition** **3.** *Information gain [24], $Gain\left(A_{i},S\right),$ is defined as:*

(3) $$Gain\left(A_{i},S\right)=E\left(S\right)-EE\left(A_{i},S\right)$$

*where S is split on an attribute*$A_{i}$.

Information gain is utilized to measure the uncertainty in S after splitting on attribute $A_{i}$.

**Definition** **4.** *The information intrinsic value [26], $IV\left(A_{i}\right),$ is defined as:*

(4) $$IV\left(A_{i}\right)=-\underset{A_{i}\in X}{\sum }p\left(A_{i}\right)\times \log_{2}p\left(A_{i}\right)$$

In the definition of the information intrinsic value, we add a parameter—the size information to $Gain\left(A_{i},S\right)$.

**Definition** **5.** *The information gain rate [26], $IGR\left(A_{i},S\right),$ is defined as follows:*

(5) $$IGR\left(A_{i},S\right)=\frac{IV\left(A_{i}\right)}{Gain\left(A_{i},S\right)}$$

Information gain is used to select the most useful attribute for classification in the ID3 DT algorithm. We calculate information gain, $Gain\left(A_{i},S\right),$ for each attribute that is not already expanded in this branch. The C4.5 algorithm modifies the information gain and proposes the concept of information gain rate to split attributes in order to overcome the shortcomings of the ID3 DT algorithm that tends to choose attributes with multiple attribute values as split attributes. It splits the set decision node into branches using the attribute for which the information gain ratio is maximum. Each new branch below the decision node presents the value of the test attribute.

## 4. Performance Assessment and Results

This section focuses on the factor evaluation based on the proposed algorithms. When we classify a mother’s degree of PPD, the PDSS Total or Short Total are used as the only indicator to perform the classification (see Figure 1). Much of the information of detail symptom descriptions are neglected. Experiments were performed considering the following four aspects:

1. Using the PDSS diagnostic questionnaire to predict the degree of PPD, what is the effect of the demographic information when we use detail symptom descriptions instead of PDSS Total or Short Total as the only indicator to perform predictions?
2. What is the preferred sequence of attributes of short form and full form to predict the degree of PPD, excluding PDSS Total and Short Total?
3. What are the relationships among the attributes of the short form and full form?
4. Can a simple rule be designed which only uses the demographic information and the short form to predict PPD effectively?

Firstly, we describe the test data and software used in our experiment and present the calculation results of the C4.5 DT in Section 4.1. We then present the data analysis based on the calculation results of the C4.5 DT and evaluate the performance from the above four aspects in Section 4.2.

### 4.1. Data Preparation and Processing

We selected the Weka machine learning algorithm to complete the factor analysis of the prediction of the PDSS. Weka contains many useful tools that can produce data preparation, classification, regression, and clustering. It can also visualize data computational results.

In this investigation, the C4.5 DT is used to build a classification model. Since our analysis involves several kinds of attributes, we studied how the classification model behaves depending on the combination of the attributes. We designed six classifiers, which are different combinations of the attributes, to define the decision tree. Our data analysis is based on two experimental test datasets. Our experiment is described below (Table 1). General calculation results of the C4.5 DT are described in Table 2. More detailed classification results of the six classifiers are separately listed in Table 3 and Table 4.

Calculating results supplied by the Weka software (see Table 5 for parameter settings) allowed us to extract several indices to create a sensitivity analysis. The sensitivity indices were defined as follows:

“Classification accuracy” is an index that describes the performance of the decision tree based on a combination of demographic information and questions of the PDSS to correctly classify all screened women (i.e., Correctly Classified Instances and Incorrectly Classified Instances).

“Confusion matrix” is the index used to describe the performance of the decision tree based on a combination of demographic information and questions of the PDSS to correctly classify according to separate classes.

“Receiver operating characteristic (ROC) area” is the area under the ROC curve that is used for evaluating classifier performance. The value of the ROC area is [0.5, 1]. The higher the number, the better the classifier performs.

**Figure 2:**
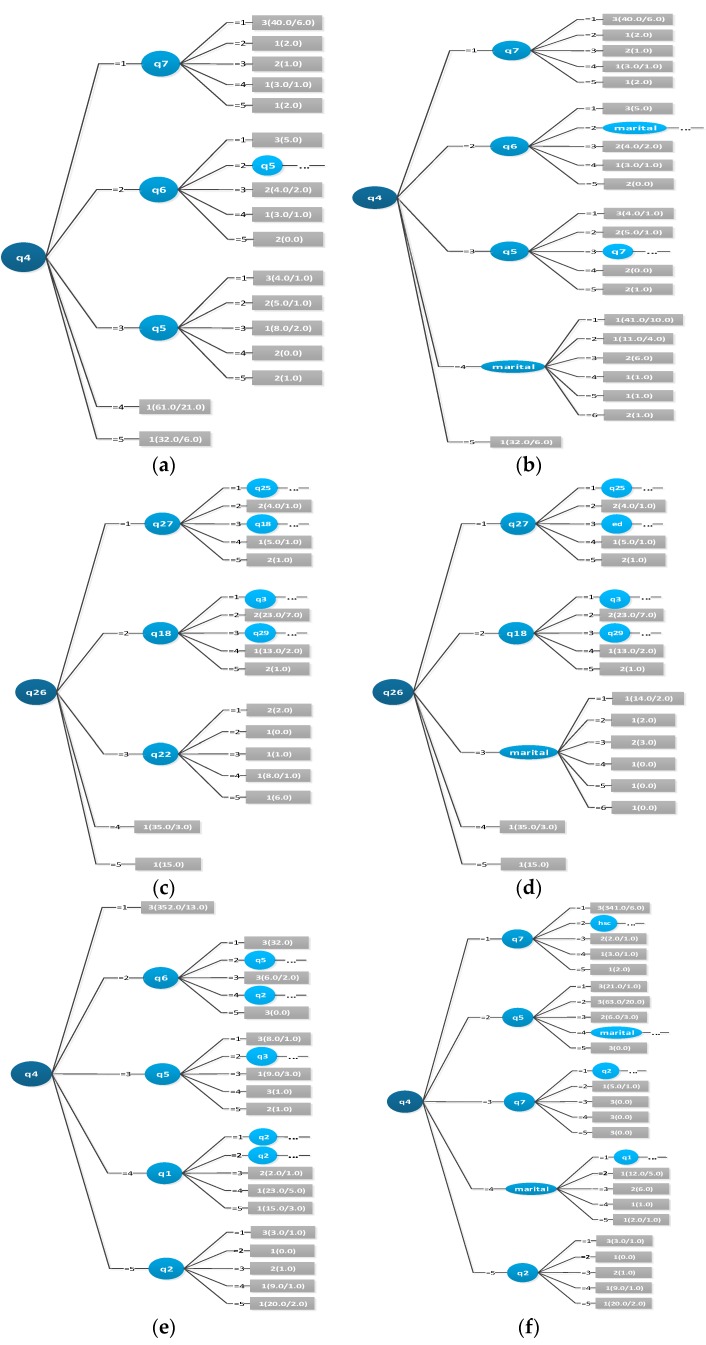
Visualized decision trees of the 6 classifiers: (**a**) the visualized decision tree of Classifier 1; (**b**) the visualized decision tree of Classifier 2; (**c**) the visualized decision tree of Classifier 3; (**d**) the visualized decision tree of Classifier 4; (**e**) the visualized decision tree of Classifier 5; (**f**) the visualized decision tree of Classifier 6.

We also used the C4.5 DT algorithm to obtain the visualized decision tree which can be used to abstract decision rules (see Figure 2). 

### 4.2. Data Analysis

After analyzing the data in Table 1, Table 2, Table 3 and Table 4, we drew the following conclusions:

1. From Table 1, we selected the indices of Precision Recall and ROC area to compare classifiers to get the rank of prediction accuracy. We can see every index of Classifier 1 is better than Classifier 2 and we conclude that Classifier 1 > Classifier 2. The Precision and Recall of Classifier 2 is better than Classifier 3, while the ROC area of Classifier 2 is worse than Classifier 3. We therefore conclude that Classifier 2 is not significantly better than Classifier 3. In the same way, we conclude that Classifier 3 is not significantly better than Classifier 4. Because Classifier 5 and Classifier 6 use different test data, we do not compare them with Classifiers 1–4. We conclude that Classifier 5 is not significantly better than Classifier 6.
2. When we use the short form to predict PPD, the seven questions combined with demographic information achieve better prediction results.
3. When we use the full form to predict PPD, the prediction results from the 35 questions combined with demographic information do not differ significantly from the results of only using the 35 questions.
4. Utilizing the confusion matrix, we can conclude that Classifier 1 is difficult to classify in the categories of PPD and classification accuracy is very low. Classifier 2 obtains better results compared to Classifier 1. Classifier 3 and Classifier 4 have no significant difference between them. Classifier 6 obtains a better result compared to Classifier 5. Any of the classifiers in Data A obtained better results when compared with any of the classifiers in Data B.
5. ROC area shows that Classifier 4 > Classifier 3 > Classifier 2 > Classifier 1 according to test Data A; and Classifier 5 > Classifier 6 according to test Data B.
6. We obtained the preferred sequence of attributes of the short form (Table 6) according to different tests after analyzing the decision tree. A visualization of the decision tree’s six classifiers is shown in Figure 2. There are some differences between the two test data sets. We conclude that the most important attributes set is { $A_{4},A_{5},A_{6},A_{7},marri\}$. In other words, we can use this set to classify PPD quickly.

### 4.3. Comparison with Other Algorithms

Moreira and Rodrigues [15] compared the performances of decision trees, support vector machines (SVMs), nearest neighbor (NN), and ensembled classifiers through biomedical and sociodemographic data analysis. Their research extracted and quantified attributes from 205 parturient women. These attributes (i.e., high blood pressure, diabetes mellitus, obesity) are different from the attributes of the PDSS. Their improved method is not suitable to compare with our method, because a classifier’s criterion selection is an important factor which decides the accuracy of a classifier. However, from the performances of these methods, we can see that psychiatric history, thyroid problems, and socioeconomic factors are important attributes to diagnose PPD. This conclusion is similar with our conclusion. We also prove that the attribute “marri” of individual demographics is in the most important attributes set. DTs can be used as an appropriate tool to predict PPD when we use detail symptom descriptions instead of the PDSS Total or Short Total as the only attribute to perform predictions.

In this section, we selected another decision tree algorithm, random forest, to classify seeing as random forest is widely used in highly correlated data sets as a non-parametric method. We also selected Weka to complete the experiment. The general calculation results of random forest are described in Table 7.

The general calculation results of the random forest DT show that the indicator of ROC Area and other methods in Moreira and Rodrigues [15] are listed in Table 8. As a high quality algorithm, random forest is no worse than another approaches in Moreira and Rodrigues [15]. Therefore, we can conclude that DT is appropriate to classify PPD.

Table 8 shows that the indicator of classification accuracy of random forest is better than the C4.5 DT algorithm. However, we still chose the C4.5 DT to predict PPD because of the following reasons. Firstly, we cannot distinguish Classifiers 1–4 in Test Data A nor distinguish Classifiers 5–6 in Test Data B. The differences between Classifier 1 and Classifier 2 are not significant. A similar situation arises with Classifier 3 and Classifier 4. We designed different classifiers to test the relationships among the factors in the PDSS questionnaire, and so the differences among pairs of Classifiers 1 and 2, pairs of Classifiers 3 and 4 and pairs of Classifiers 5 and 6 should be larger. There are also no differences between Classifier 5 and Classifier 6 from Table 6. Although the classification accuracy of the random forest DT is better than the C4.5 DT algorithm, we cannot do deep analysis of the relationship among attributes, and therefore did not use it as an algorithm.

## 5. Discussion

According to the instructions on interpreting the PDSS, women have to enter their demographic information on the summary sheet and then complete the short form. Questions 1–7 in the short form have to be completed. If the PDSS short score total is ≤13, the woman does not need to be referred for mental health evaluation at this time. Our analysis of the 586 questionnaires showed a PDSS short score total ≤13 in 382 instances, while 29 out of the 382 instances still completed the full form. In the other 204 instances, the PDSS short score total was >13, and 24 of these did not complete the PDSS full form. Our Test Data B contains all 586 instances which is composed of 382 instances whose PDSS short score total is ≤13 and 204 instances whose PDSS short score total is >13. The Test Data B furthermore contains 209 instances of completed short forms and full forms.

The degree of a mother’s PPD can be assessed according to the scores of the PDSS full total and short total. The 35 symptom scales are replaced by one score; is easy to operate. However, much of the information has been neglected in previous studies. Additionally, some studies [18,19,20,21] examined the role of socioeconomic variables in screening practices; however, these studies were not based on the PDSS questionnaire. Our results are obtained from a tangible pregnancy database based on the PDSS questionnaire.

We designed six classifiers to map the relationships from attribute scores to categories. We removed the Short Total and PDSS Total in the six classifiers to reduce their impact on categories and highlighted the role of other attributes. Due to adding the impact of demographic information (11 attributes), Correctly Classified Instances of Classifier 2 improved compared to Classifier 3. The improvement of Classifier 2 was, however, not significant when compared with Classifier 3. The 11 attributes of demographic information can help detect depression even when women only complete the short form. If we consider the 11 attributes of demographic information when we detect PPD, the other 28 attributes of the full form cannot improve the quality of the PPD diagnosis. We can therefore conclude that in the case of limited information, seven questions from the short form combined with 11 attributes of demographic information can help us obtain effective diagnosis results. Even when we distinguish the first and the second categories, correctly classified ratios are better compared than when only using the 28 items from the full form.

By extracting the rules after generation of the decision tree, we establish the rank of the attributes. We select the five most important attributes and calculate their $Gain\left(F_{i},S\right)$ (see Table 5). A set of attributes is described as follows:

- $A_{4}$ is the most important attribute when the information is limited. The Inconsistent Responding Index (INC) has to calculate the difference between $A_{4}$ and $A_{18}$. $A_{4}$ is described as “I felt like I was losing my mind.” $A_{18}$ is described as “I thought I was going crazy.” These two questions are asked to determine the similar diagnostic symptom of PPD.
- $A_{26}$ is the most important attribute when the full form is completed. INC has to calculate the difference between $A_{5}$ and $A_{26\;}.$ $A_{5}$ is described as “I was afraid that I would never be my normal self again.” $A_{26}$ is described as “I felt like I was not normal.” These two questions are asked to obtain the similar diagnostic symptom of depression.
- Marital status is an attribute from demographics information. There are not any corresponding attributes in the full form or short form. The combination of this attribute with other attributes can achieve better prediction of PPD.

## 6. Conclusions

Through the analysis of 586 samples of the PDSS questionnaire, collected from Robeson County in North Carolina, we have determined the role of attributes in the PDSS as well as the rules for using these attributes to classify PPD. An important contribution of this paper is that an actual pregnancy database was used to verify our conclusion. There still are some limitations, nonetheless. The seven dimensions and five questions for each dimension are designed to correspond to the diagnostic symptoms.

One limitation of the study is assumptions. We assume that the factors used to predict PPD are independent. In other words, that the seven dimensions: Sleeping/Eating Disturbances, Anxiety/Insecurity, Emotional Lability, Cognitive Impairment, Loss of Self, Guilt/Shame, and Contemplating Harming Oneself are independent. However, the symptoms are not independent at all. For example, weight loss, body image change, work difficulty, and somatic preoccupation may be related. Somatic preoccupation may be the result of or reason for weight loss, body image change, or work difficulty. We should therefore pay attention to these interactions that can influence our predictions. Further research on the PDSS should also consider the interactions between the attributes on PDSS. Non-additive fuzzy measure, which is an appropriate tool to solve the problem of interactions among criteria, can be used to aggregate attributes’ evaluations. 

The other limitation is the use of labels from (1) to (5) to reflect the respondent’s degree of disagreement or agreement. When completing the scale, a mother is asked to select a label. The mother selects a word to express her preference, but her preference is a fuzzy concept. In order to simplify the prediction process, a crisp number is used to replace a word. However, this replacement is not appropriate. The technique of Computing With Word [27] can be used to reflect a mother’s preference accurately. Information loss can thus be avoided in future research.

## Figures and Tables

**Table 1:** Description of the experiment.

	Description of Test Data	Demographics Information	Questions 1–7 (Labeled A_1_–A_7_)	Questions 8–35 (Labeled A_8_–A_35_)	
Test data A	Contains 209 instances which are abstracted from “PPD 2006 Jan ALL 586 records” ^1^		✓		Test 1/classifier 1
		✓	✓		Test 2/classifier 2
			✓	✓	Test 3/classifier 3
		✓	✓	✓	Test 4/classifier 4
Test data B	PPD 2006 Jan ALL 586 records		✓		Test 5/classifier 5
		✓	✓		Test 6/classifier 6

^1^ Full scale scores of the 209 instances are all 1, which means these instances have completed both the short form and full form.

**Table 2:** General calculation results of the C4.5 decision tree (DT).

	Number of Attributes	Correctly Classified Instances (n)	Correctly Classified Instances (%)	Incorrectly Classified Instances (n)	Incorrectly Classified Instances (%)	Precision	Recall	ROC Area
Test 1 (Classifier 1)	7	140	66.98	69	33.01	0.654	0.670	0.757
Test 2 (Classifier 2)	18	150	71.77	59	28.33	0.712	0.718	0.771
Test 3 (Classifier 3)	35	146	69.86	63	30.14	0.701	0.699	0.794
Test 4 (Classifier 4)	46	146	69.86	63	30.14	0.698	0.699	0.799
Test 5 (Classifier 5)	7	480	81.91	106	18.09	0.789	0.819	0.852
Test 6 (Classifier 6)	18	486	82.94	100	17.06	0.792	0.829	0.847

**Table 3:** Confusion matrix of the four classifiers of Test Data A.

	A	B	C	Classified as
Test 1	77	10	1	1
	29	20	11	2
	6	12	43	3
Test 2	78	9	1	1
	21	29	10	2
	6	12	43	3
Test 3	68	14	6	1
	14	33	13	2
	2	14	45	3
Test 4	69	14	5	1
	15	32	13	2
	3	13	45	3

**Table 4:** Confusion matrix of the two classifiers of Test Data B.

	A	B	C	Classified as
Test 5	63	8	17	1
	21	7	32	2
	17	11	410	3
Test 6	66	8	14	1
	23	5	32	2
	14	9	415	3

**Table 5:** Parameter settings.

Pre-Process	Attributes Filters	Numerical to Nominal
Classify	Cross-validation	10-fold cross-validation
	Classifier	J48

**Table 6:** The preferred sequence of attributes of the short form.

	i	$\;Gain\left(A_{i},S\right)\;$	i	$\;IG\left(A_{i},S\right)\;$	i	$\;IG\left(A_{i},S\right)\;$	i	$\;IG\left(A_{i},S\right)\;$	*i*	$\;IG\left(A_{i},S\right)\;$
Test Data A	4	0.314	5	0.279	6	0.259	7	0.200	marri	0.017
Test Data B	4	0.388	7	0.313	6	0.277	5	0.185	marri	0.006

**Table 7:** The general calculation results of random forest DT.

	Number of Attributes	Correctly Classified Instances (n)	Correctly Classified Instances (%)	Incorrectly Classified Instances (n)	Incorrectly Classified Instances (%)	Precision	Recall	ROC Area
Test 1 (Classifier 1)	7	143	68.42	66	31.58	0.677	0.684	0.851
Test 2 (Classifier 2)	18	142	67.94	67	31.06	0.664	0.679	0.848
Test 3 (Classifier 3)	35	169	80.86	40	19.14	0.801	0.809	0.941
Test 4 (Classifier 4)	46	163	77.99	46	22.01	0.771	0.780	0.927
Test 5 (Classifier 5)	7	506	86.35	80	13.65	0.840	0.863	0.958
Test 6 (Classifier 6)	18	506	86.35	80	13.65	0.840	0.863	0.958

**Table 8:** Comparison of indicator ROC Area ^1^.

Approach	ROC Area
Simple Tree	0.907
Linear SVM	0.912
Weighted k-Nearest Neighbor (kNN)	0.865
Bagged Trees	0.914
Random forest DT	0.914
C4.5 DT	0.803

SVM = support vector machine. ^1^ The value of random forest DT and C4.5 DT ROC Area is the average of the 6 classifiers in Table 6 and Table 2. The value of other the four approach’s ROC Area is the average of Table 3 in [15].
